# An overview of malaria transmission from the perspective of Amazon *Anopheles vectors*

**DOI:** 10.1590/0074-02760140266

**Published:** 2015-02

**Authors:** Paulo FP Pimenta, Alessandra S Orfano, Ana C Bahia, Ana PM Duarte, Claudia M Ríos-Velásquez, Fabrício F Melo, Felipe AC Pessoa, Giselle A Oliveira, Keillen MM Campos, Luis Martínez Villegas, Nilton Barnabé Rodrigues, Rafael Nacif-Pimenta, Rejane C Simões, Wuelton M Monteiro, Rogerio Amino, Yara M Traub-Cseko, José BP Lima, Maria GV Barbosa, Marcus VG Lacerda, Wanderli P Tadei, Nágila FC Secundino

**Affiliations:** 1 Centro de Pesquisas René Rachou-Fiocruz, Belo Horizonte, MG, Brasil; 2 Fundação de Medicina Tropical Dr Heitor Vieira Dourado, Manaus, AM, Brasil; 3 Instituto Oswaldo Cruz-Fiocruz, Rio de Janeiro, RJ, Brasil; 4 Instituto Leônidas e Maria Deane-Fiocruz, Manaus, AM, Brasil; 5 Instituto Nacional de Pesquisas da Amazônia, Manaus, AM, Brasil; 6 Unité de Biologie et Génétique du Paludisme, Institut Pasteur, Paris, France

**Keywords:** Anopheles, Plasmodium, transmission, Amazon vectors

## Abstract

In the Americas, areas with a high risk of malaria transmission are mainly located in the Amazon Forest, which extends across nine countries. One keystone step to understanding the Plasmodium life cycle in Anopheles species from the Amazon Region is to obtain experimentally infected mosquito vectors. Several attempts to colonise Ano- pheles species have been conducted, but with only short-lived success or no success at all. In this review, we review the literature on malaria transmission from the perspective of its Amazon vectors. Currently, it is possible to develop experimental Plasmodium vivax infection of the colonised and field-captured vectors in laboratories located close to Amazonian endemic areas. We are also reviewing studies related to the immune response to P. vivax infection of Anopheles aquasalis, a coastal mosquito species. Finally, we discuss the importance of the modulation of Plasmodium infection by the vector microbiota and also consider the anopheline genomes. The establishment of experimental mosquito infections with Plasmodium falciparum, Plasmodium yoelii and Plasmodium berghei parasites that could provide interesting models for studying malaria in the Amazonian scenario is important. Understanding the molecular mechanisms involved in the development of the parasites in New World vectors is crucial in order to better determine the interaction process and vectorial competence.

Malaria is an infectious disease that has a major impact on global public health and the economy, with an estimated 3.4 billion people at risk. Currently, malaria threatens almost one third of the world's population in 104 tropical countries and territories where it is considered an endemic disease. The World Health Organization (WHO) estimates that 207 million cases of malaria occurred globally in 2012 and led to 627,000 deaths. Africa, South-East Asia and the Eastern Mediterranean were the regions with the highest numbers of reported cases and deaths reported, mainly in children under five years of age (WHO 2013).

In the Americas, 22 countries are affected by malaria, with approximately 1.1 million cases and 1,100 deaths registered in 2010. In this continent, 30% of the population is considered to be at risk and 8% are classified as being at high risk. Areas with a high transmission risk are mainly located in the Amazonian rainforest, which extends across nine countries including Brazil, Bolivia, Colombia, Ecuador, Peru, Venezuela, Guyana, Suriname and French Guiana. Brazil and Colombia accounted for 68% of the malaria cases in 2011 (PAHO 2011, WHO 2013).

In Brazil, approximately 241,000 clinical cases and 64 deaths were registered in 2012, most of them (99.88%) in the Amazon Region where malaria is endemic in nine states, namely, Acre, Amapá (AP), Amazonas (AM), Mato Grosso, Pará (PA), Rondônia, Roraima, Tocantins and Maranhão. PA and AM registered almost 70% of the cases in 2012; 14.4% were in urban areas, 25% in gold mine exploitation areas and the others were in rural settlements and indigenous areas (MS/SVS 2013, SVS 2013).

A gradual reduction in the overall number of cases has been observed over the last five years, but there has also been a significant increase in the number of cases in the Brazilian Amazon Region in 2012. Factors that contributed to the increased transmission of malaria include intensive and disorganised occupancy on the outskirts of cities, deforestation and artificial fishponds (MS/SVS 2013, SVS 2013).

Outside the Amazon Region, there were 914 cases registered in 2012 in different Brazilian states, mainly in São Paulo (SP) (188), Rio de Janeiro (130), Minas Gerais (105), Goiás (82) and Piauí (72) (SVS 2013 ). Most of these cases were due to migration from the Amazon Region or from the African continent, but a few were autochthonous from the endemic Atlantic Forest endemic region where few foci are maintained (Rezende et al. 2009, Duarte et al. 2013, Neves et al. 2013).

Malaria is due to infection by a parasitic protozoa of the *Plasmodium *genus. Several *Plasmodium *species infect humans and other animals, including birds, reptiles and rodents. In Brazil, three human *Plasmodium* parasites are prevalent. *Plasmodium vivax* is the predominant species (83.81%) and is responsible for cases associated with severe clinical complications and death (Alexandre et al. 2010, Costa et al. 2012, Lacerda et al. 2012). The prevalence of *Plasmodium* *falciparum* (13.15%) has declined in the last decade, whilst *Plasmodium malariae* is the least prevalent species (0.037%). However, these numbers may be underestimated because the thick blood smear method that is used for routine malaria diagnosis may lead to misidentification of the species (Cavasini et al. 2000).

## Plasmodium cycle in the vector 

Mosquitoes of the *Anopheles* genus are the vectors of the *Plasmodium* species, the causative agents of malarial disease. More than 400 species of the *Anopheles* mosquito have been described and approximately 70 these species are potential vectors of malaria that affect humans (Sinka et al. 2012 ). In the natural vector, the life cycle starts when the female *Anopheles *mosquito takes a blood meal from an infected vertebrate host and ingests gametocytic forms of the parasite that are present in the blood (Smith et al. 2014).

One mosquito ingests an average of 10^3 ^gametocytes in an infected blood meal. Within minutes after the infective blood meal, these gametocytes undergo maturation inside the lumen of the midgut, which generates micro and macrogametocytes that will be fertilised and produce a diploid zygote (Sinden 1999 ). The mature zygote will differentiate into the mobile form of the parasite known as the ookinete *via* a process that can take up to 16-24 h, depending on the *Plasmodium *species (Ghosh et al. 2000, Dinglasan et al. 2009). This process starts with the exflagellation of the gametocytes in the mosquito's midgut after ingestion of the infected blood meal. Exflagellation will lead to the formation of the micro and macrogametocytes and occurs mainly due to differences in temperature and pH and the production of xanturenic acid by the mosquito (Billker et al. 1997 , 1998). The formation of the zygote occurs after fertilisation of the micro and macrogametocytes and will eventually differentiate into an ookinete. This development will only occur if the parasites are able to defeat the action of the digestive enzymes that are secreted by the epithelium and are active throughout the midgut. It is believed that the ookinetes in the outer parts of the blood meal will die first from the actions of these digestive enzymes and the ookinetes that are closer to the interior of the blood meal and consequently farther away from the effects of the enzyme, will have a longer time in which to differentiate and survive the actions of the enzyme (Abraham & Jacobs-Lorena 2004). The ookinete, which is the mobile form of the parasite, will move and penetrate the peritrophic matrix (PM) and pass through the intestinal epithelium before transforming into an oocyst (Smith et al. 2014).

**Fig. 1:**
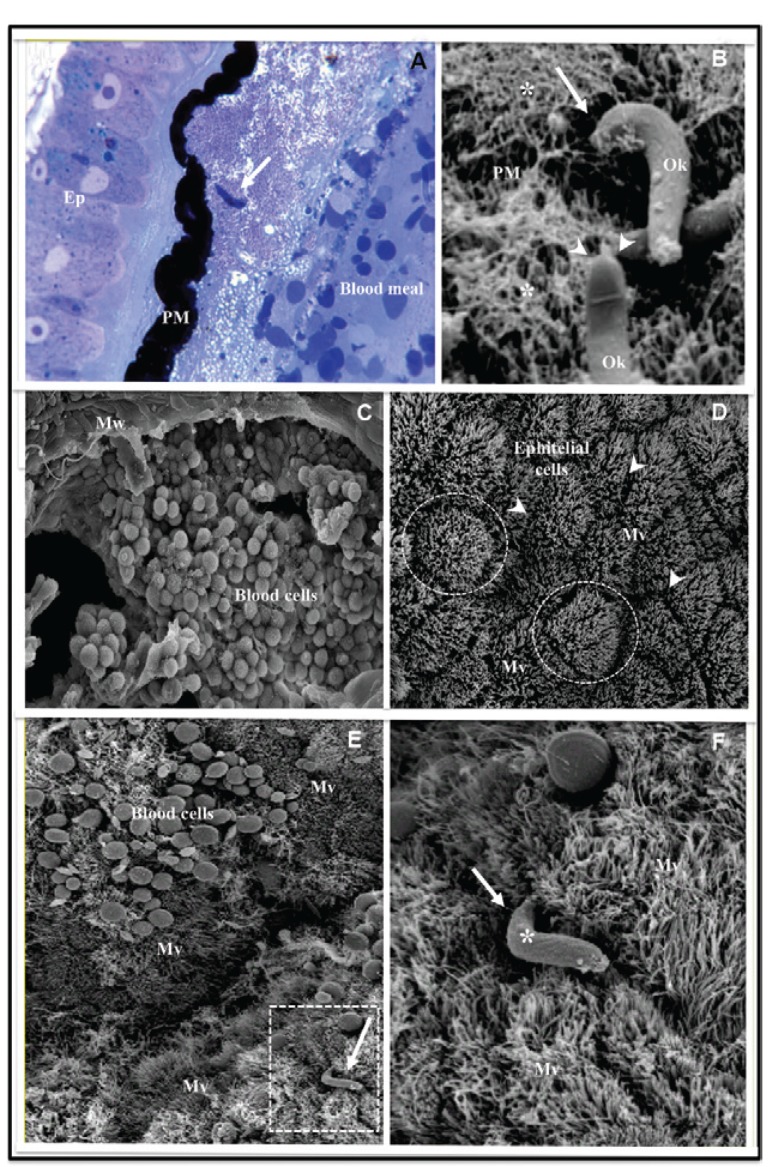
histology (A) and scanning electron microscopy (SEM) (B-F) of Anopheles aquasalis midguts after a Plasmodium vivax infective blood meal. A: historesin section of a midgut stained with Giemsa. The peritrophic matrix (PM) sturdily stained in black is separating the midgut epithelium (Ep) from the blood meal. Note an ookinete (Ok) (arrow) close to the PM; B: SEM of an opened midgut showing two Oks over the PM. Observe the fibrous aspect (asterisks) of the internal side of the PM. One Ok is crossing the PM throughout the fibre layer (large arrow). Another Ok is showing details of its anterior extremity (arrowheads); C: small magnification of an opened midgut showing the blood meal containing the numerous blood cells. Note a portion of the midgut wall (Mw); D: large magnification of an opened midgut showing details of the epithelial cells. The epithelial cells have polygonal shapes (circles) and their surfaces are covered by microvilli (Mv). Note the clefts (arrowheads) among the epithelial cells; E: small magnification of an opened midgut with blood cells of the blood meal. Note inside the square area one Ok (arrow) penetrating the Ep Mv; F: large magnification of the square area of E in the Figure showing details of the Ok penetration. Note the Ok (asterisk) extremity inserted in a cleft (asterisk) among the epithelial cell Mv.

The PM is a layer comprised of chitin, proteins and proteoglycans that surround the blood meal that has been ingested (Fig. 1). Physical distension caused by the ingestion of the blood and the blood meal itself are signals for the mosquito's midgut to induce the formation of the PM. This matrix is seen as a physical barrier to many parasites as it prevents their contact with the insect gut (Ghosh et al. 2000). Several studies have suggested that *P. falciparum* and *Plasmodium gallinaceum* may secrete chitinase additional to that already produced by the insect which would allow the parasite to accomplish three crucial steps in the infection of the invertebrate host: (i) penetrate through the PM, (ii) escape the deadly action of digestive enzymes and (iii) successfully invade the epithelial cells of the intestine (Huber et al. 1991, Dessens et al. 1999, Vinetz et al. 1999, 2000). The details of the penetration of the PM by the ookinete are seen in Fig. 1A, B. The recently transformed ookinete moves in the direction of the mosquito epithelium (Fig. 1A) and penetrates the PM by introducing its anterior extremity into the fibrous layer of the internal side of the PM (Fig. 1B).

The penetration of the *Plasmodium* ookinete into the midgut epithelium is an important step in the infection of mosquitoes and has been thoroughly studied previously (Fig. 1B-F). The epithelial cells have polygonal shapes and their surfaces are covered with microvilli (Fig. 1D). The ookinete penetrates the microvilli clefts that exist among the epithelial cells toward their anterior extremity (Fig. 1E, F) in order to initiate the invasion process.

Different theories have arisen regarding the ookinete's strategies for penetration and invasion of the epithelial cells and escaping detection by the host's immune system. After several years without any conclusive studies on how the ookinete invades the mosquito epithelium, Shahabuddin and Pimenta (1998) used an in vitro system to study the interaction of *P.* *gallinaceum* with *Aedes aegypti*. The methodology consisting of the incubation of the parasites with dissected midgut was successfully applied to a study of the *Leishmania-*vector interaction (Pimenta et al. 1992, 1994). The result suggested the existence of specialised cells in the midgut epithelium of *Ae. aegypti* that the authors called Ross cells, which would serve as a specific entry point for the ookinete (Shahabuddin & Pimenta 1998). Subsequently, Han et al. (2000) proposed a time bomb theory in which parasites invade any epithelial cell in the midgut and this process of penetration triggers an immune response, causing this particular cell to begin apoptosis. However, a conclusive report from Barillas-Mury's group at National Institute of Allergy and Infectious Diseases that was completed with our collaboration (Gupta et al. 2005 ) indicated that *Ae. aegypti *and *Anopheles stephensi *differ in their mechanisms of epithelial repair after *Plasmodium* ookinete invasion.* An. stephensi *damaged cells *via* an actin-mediated budding-off mechanism when invaded by either *Plasmodium berghei *or *P. gallinaceum*. In *Ae. aegypti*, the midgut epithelium is repaired by a unique actin cone zipper mechanism that involves the formation of a cone-shaped actin aggregate at the base of the cell that closes sequentially, expelling the cellular contents into the midgut lumen as it brings together healthy neighbouring cells. This study had important findings: (i) it determined that the apparent target cells used by *P. gallinaceum* to invade the vector epithelium were in fact an in vitro artifact; the Ross cells are believed to represent cells that have lost their integrity and some of their cytoplasmic contents after parasite invasion and (ii) these studies indicated that the epithelial responses of different mosquito vectors to *Plasmodium *depend on the vector-parasite combinations and are not universal.

**Fig. 2:**
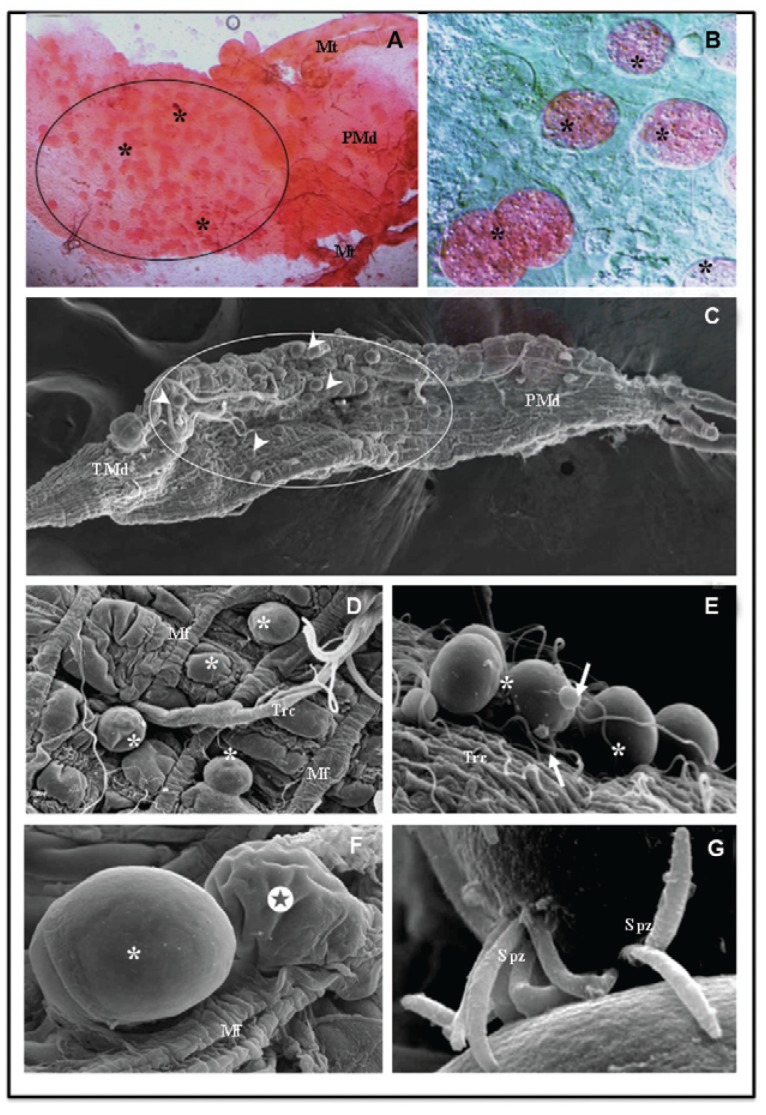
optical microscopy (OM) and scanning electron microscopy (SEM) of Anopheles aquasalis midguts infected with Plasmodium vivax. A: small magnification of a dissected infected midgut stained with commercial mercurochrome and visualised by an OM. Note in the elliptical area the presence of numerous oocysts (asterisks); B: large magnification image of the A in Figure. Observe the granular aspects of the developing rounded oocysts (asterisks) in the midgut wall; C: SEM small magnification image of a dissected infected midgut. Note inside the elliptical area the presence of several rounded oocysts (arrowheads) protruding from the midgut wall. The oocysts are concentrated in the transition region between the thoracic midgut (TMd) and the posterior midgut (PMd); D: SEM image of oocysts (asterisks) protruding among the microfibres (Mf) that are presenting outside the midgut wall; E: a group of oocysts (asterisks) are seen protruding on the midgut wall. They are surrounding by small tracheoles (Trc). Two haemocytes (arrows) are attached to one oocyst; F: a large magnification view of two oocysts showing one with a smooth surface (asterisk) and another with shrunk surface (black star) possibly due to the liberation of sporozoites (Spz) into the haemocoel; G: large magnification of SEM images of a group of Spz that already escaped from the oocysts and are free in the mosquito haemocoel; Mt: Malpighian tubules.

After crossing the epithelial layer of the gut, the ookinetes will remain between the intestinal epithelium and the basal lamina, at which point the maturation of the oocyst will occur. A simple method of staining with mercurochrome (Merbromin) solution is useful for the identification of infected midguts. The rounded oocysts can be seen in bright red (Fig. 2A, B). Scanned electron microscope images of the external side of the infected midguts are valuable for showing the morphological aspects of the developing oocysts (Fig. 2C-F). These oocysts appear as protruding structures among the muscle fibres of the midgut wall (Fig. 2D). Some haemocytes can be seen attached to oocysts (Fig. 2E). It is also possible to observe shrunken oocysts due to the rupture of the oocyst wall (Fig. 2F). Oocyst rupture and the subsequent release of sporozoites occur once the maturation is complete (usually within 10-24 days, depending on the *Plasmodium *species). This leads to the release of anywhere from hundreds to thousands of sporozoites into the mosquito haemocoel (Hillyer et al. 2007 ) (Fig. 1G). Before reaching the salivary gland, the sporozoites still need to overcome the other barriers that is produced by the immune system, including: (i) haemocytes (Fig. 2E), which are cells that are responsible for the internal defense system of the mosquito, (ii) antimicrobial peptides and (iii) other humoral factors (Dimopoulos et al. 2001 ).

In general, the process of invasion of the salivary gland by sporozoites is very inefficient; usually less than 20% of the total numbers of parasites produced are able to invade the organ (Korochkina et al. 2006, Hillyer et al. 2007). Those sporozoites that survive after overcoming various barriers to reaching the salivary gland are finally able to invade the organ. By means of a specific recognition receptor present in the salivary gland of the vector, these parasites are able to adhere to and penetrate the basal lamina of the gland before penetrating the host plasma membrane of the salivary cells. A number of parasite ligands are necessary for the initial attachment of the sporozoites to the salivary glands, such as some regions of the circumsporozoite protein and thrombospondin-related anonymous protein [see details in Sinden and Matuschewski (2005) and Aly et al. (2009)]. This process of invasion has been well described using the *P. gallinaceum*/*Ae. aegypti *model (Pimenta et al. 1994). The penetration process appears to involve the formation of membrane junctions. Once inside the host cells, the sporozoites are seen within vacuoles attached by their anterior end to the vacuolar membrane. Mitochondria surround and are closely associated with the invading sporozoites. After the disruption of the membrane vacuole, the parasites traverse the cytoplasm, attach to and invade the secretory cavity through the apical plasma membrane of the cells. Inside the secretory cavity, the sporozoites are again seen inside the vacuoles. Upon escaping from these vacuoles, the sporozoites are positioned in parallel arrays, forming large bundles attached by multilamellar membrane junctions. Several sporozoites are seen inside and around the secretory duct. Except for the penetration of the chitinous salivary duct, these observations have morphologically characterised the entire process of sporozoite passage through the salivary gland (Pimenta et al. 1994). The sporozoites that are now inside the secretory duct of the salivary gland are ready to be injected by the mosquito bite into the skin of a new vertebrate host. An analysis of the amount of parasite that an infected mosquito could inject into the skin of a mouse varied between zero and approximately 1,300 and there appears to be a weak correlation of the number of injected sporozoites with the salivary gland load (Medica & Sinnis 2005).

**Fig. 3:**
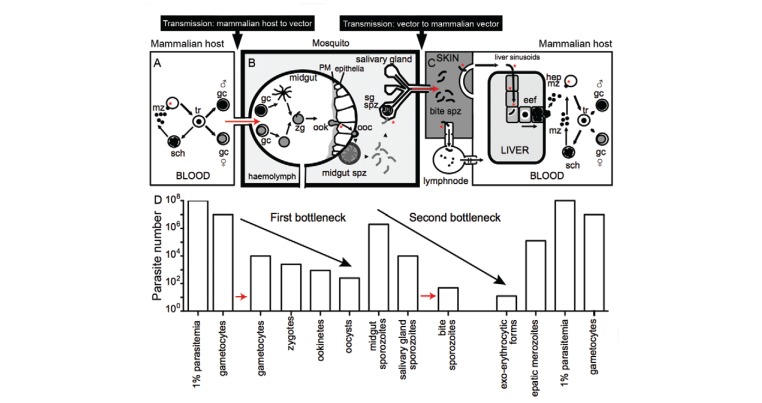
parasite load inside the vertebrate and invertebrate hosts. Qualitative view of the major steps in the life-cycle of Plasmodium parasites inside the mammalian host (A-C) and the mosquito vector (B). Invasive steps are marked with a red asterisks and parasite transmission by red arrows. A: merozoites (mz) invade red blood cells (RBCs) and transform in trophozoites (tr). After asexual division, tr mature in schizonts (sch), which liberate new mz in the blood circulation. Some mz can also differentiate into male or female gametocytes (gc) inside infected RBCs; B: these sexual dimorphic stages are ingested by a mosquito during a blood meal and after activation reproduce sexually generating a zygote (zg). The zg differentiates into the motile ookinete (ook) that crosses the peritrophic matrix (PM) and midgut epithelial cells to develop as an oocyst (ooc) in the laminal basal of the midgut. The ooc then generates midgut sporozoites (spz) that after being released into the haemolymph, invade and are stored in the mosquito salivary glands (sg); C: during the bite the infected mosquito deposits spz (bite spz) in the extravascular parts of the skin. Some spz invade lymph vessels, but are trapped and degraded in the draining lymph nodes. Some spz invade blood vessels and reach the liver sinusoids. After invading the liver parenchyma and traversing host cells, the spz invades and develops as an exoerythrocytic form (eef) in a parasitophorous vacuole inside a hepatocyte. The eef generates hepatic mz (hep mz) that are released inside merosomes in the blood circulation initiating a new cycle of RBC invasion; D: quantitative view of the major steps in the life-cycle of Plasmodium parasites. The bars represent the estimated number of Plasmodium berghei parasites infecting mice and Anopheles stephensi mosquitoes. Data modified from Baton and Ranford-Cartwright (2005), Medica and Sinnis (2005), Amino et al. (2006) and Sinden et al. (2007). Parameters for estimation: 1e10 RBCs/mouse, 1 μL of blood ingested by mosquito, ratio 1 gametocyte: 10 infected RBC, 25% of bite spz infect hepatocyted, 1 eef generates 10,000 hep mz.

Considering the entire *Plasmodium* life cycle in the vector and in the vertebrate host, it is fascinating to observe the complexity of distinct developmental forms and the parasite load during the course of infection. There is extraordinary adaptation of the *Plasmodium* parasite to its environment, which is reflected in morphological changes and the parasite load of distinct organs inside the vertebrate host and the mosquito vector (Baton & Ranford-Cartwright 2005 , Medica & Sinnis 2005, Amino et al. 2006, Ma et al. 2010 , Smith et al. 2014). During the stages that the *Plasmodium* moves from the mammalian host to the vector and *vice versa*, two "bottlenecks" occur that are characterised by a small number of parasites. Fig. 3 shows an animated model that illustrates qualitative and quantitative views of the major steps of the life cycle of the *P. berghei* parasites infecting mice and *An. stephensi* mosquitoes. Murine-*Plasmodium *spp interaction studies are considered to be suitable experimental models to better understand the interaction between malarial parasites and vectors.

## The key Amazon Anopheles vectors

Among the *Anopheles *mosquito species that inhabit the Amazon, *Anopheles* *darlingi, Anopheles albitarsis* *s.l.* and* Anopheles aquasalis* are considered the principle mosquito vectors*. *Specifically*, An. darlingi* is the main vector in South America and has been associated with the dynamics of malaria transmission in the Amazonian regions of Bolivia, Colombia, French Guiana, Guyana, Peru, Suriname and Venezuela (Zimmerman 1992, Hiwat et al. 2010). *An. albitarsis s.l. *inhabits regions of Venezuela (Rubio-Palis et al. 1992) and *An. aquasalis* is found in Trinidad (Chadee & Kitron 1999 ), Guyana (Laubach et al. 2001) and Venezuela (Berti et al. 1993).

Other anopheline species can be secondary or occasional malaria vectors because of their population density, anthropophilic behaviour and natural infectivity across their geographical distributions (Deane 1986 , Zimmerman 1992, Sinka et al. 2010, 2012). *Anopheles nuneztovari s.l*. and *Anopheles triannulatus s.l.* are commonly collected in the Amazon by researchers and they have been observed to be infected with *P. vivax* and *P. falciparum*, but their role as malaria vectors has yet to be elucidated (de Arruda et al. 1986, de Oliveira-Ferreira et al. 1990, Klein et al. 1991 b, Tadei & Dutary 2000, da Silva-Vasconcelos et al. 2002 , Póvoa et al. 2003, 2006, dos Santos et al. 2005 , Galardo et al. 2007, da Rocha et al. 2008 , Santos et al. 2009).

Recently, Foley et al. (2014) developed a study considering the percentage of the area predicted to be suitable for mosquito habitation based on ecological niche models of Amazon vectors. They found that *An. albitarsis *I, *Anopheles janconnae* and *Anopheles marajoara *had the highest percentage of their predicted suitable habitats overlapping the distribution models of *P. falciparum *and *P. vivax *[see details in Foley et al. (2014)]. They also concluded that phylogenetic proximity might be related to malaria vectorial importance within the *Albitarsis* group. The authors recognised that these findings would encourage additional studies of the transmission potential of these Amazonian *Anopheles* species.

*An. aquasalis* is distributed predominantly along the Atlantic Coast because of its tolerance to saltwater environments, including in Venezuela, where it is considered to be the primary coastal malaria vector of *P. vivax *(Galvão et al. 1942, Laubach et al. 2001, Póvoa et al. 2003, da Silva et al. 2006a).

Amazonian *Anopheles* species such as*, Anopheles deaneorum, An. marajoara, Anopheles mattogrossensis, An. nuneztovari, Anopheles oswaldoi, Anopheles rondoni* and* An. triannulatus *have been considered "naturally infected" with *Plasmodium* since they were captured with parasites in their blood meal (Galvão et al. 1942, Deane et al. 1948, de Arruda et al. 1986, Klein et al. 1991b, Branquinho et al. 1993, Tadei & Dutary 2000, Póvoa et al. 2001, 2003, 2006, da Silva-Vasconcelos et al. 2002, da Silva et al. 2006a, Galardo et al. 2007, da Rocha et al. 2008, Santos et al. 2009). However, their role as malaria vectors is not well defined.

Two crucial factors needed to label a mosquito a vector are the demonstration that the species is anthropophilic and identification of the same *Plasmodium *species or strain in patients from the same geographic region. In the field, the presence of *Plasmodium *oocysts in the mosquito midgut indicates parasite establishment in a susceptible vector. However, the discovery of only sporozoites in the dissected mosquito salivary gland can confirm that the life cycle is complete and consequently that the *Plasmodium* parasite can be transmitted by a bite to human hosts. Moreover, recognition of the infection rate (i.e., the percentage of individuals in a mosquito population that carry *Plasmodium*) is an important parameter for defining vector competence and thus a key indicator in the description of malaria dynamics and transmission biology in a given geographic region. In contrast, the sole presence of an apparent abundance of a species along with parasites in the ingested blood meal is not sufficient to implicate a mosquito as a vector (Smith et al. 2014 ).

## Colonisation of American anophelines 

Considering *An. darlingi*, *An. albitarsis* *s.l. *and *An. aquasalis is *as main vectors*, *only the latter species has been colonised for several years under laboratory conditions (Lima et al. 2004). The maintenance of mosquito vectors in a laboratory facilitates studies on their biology and behaviour and experimental studies to characterise details of their susceptibility to *Plasmodium* species, thus providing a greater understanding of malaria disease dynamics. Mosquito vectors of malaria from Africa and Asia have been well established in colonies and can be maintained in insectaries of several laboratories in different countries. Consequently, *Anopheles gambiae*, the major vector in several African countries, is the most well studied mosquito, including its interaction with human and murine *Plasmodium *species that are considered causative agents of malaria (Moores 1953). Distinctly, the colonisation of *An. darlingi*, the major Amazon vector, has proven to be difficult, as has that of other New World anopheline species.

Several attempts to colonise American species of *Anopheles* under laboratory conditions have been conducted either unsuccessfully or with short-lived success. When describing the rationale for establishing a colony of *Anopheles* *quadrimaculatus,* Boyd et al. (1935) highlighted two key starting points: (i) an abundant supply of food for the larvae and (ii) a stable and optimal temperature. Galvão et al. (1944) used Boyd's technique with specifically sized cages (40 x 40 x 47 cm). They loaded approximately two thousand mosquitoes into each cage and the females started to lay eggs after seven days. Reproduction led to *An.* *albitarsis domesticus* (*An.* *marajoara*) mosquitoes reaching the seventh generation. Egg production in *Anopheles* *tarsimaculatus* (*An.* *aquasalis*), however, was low and was maintained by only a few dozen couples up to the fifth generation. The authors attributed the colonisation problems to a lack of mating due to the space and type of food offered to the males. To begin a mosquito colony there are numerous factors that need to be controlled for, including the fact that several species do not undergo free copulation under laboratory conditions (Martinez-Palacios & Davidson 1967). Thus, for the establishment of the colony, the induced copulation approach is often necessary. This method was developed by McDaniel and Horsfall (1957) for the *Aedes* spp and was later adapted by Baker et al. (1962) for *Anopheles*.

There are descriptions in the literature of various American *Anopheles *species that have been maintained in insectaries for short periods of time, including *Anopheles* *punctipennis*,* Anopheles* *maculatus*, *An.* *aquasalis,* *An.* *albitarsis*,* An.* *deaneorum* and *An.* *marajoara* (Baker et al. 1962, Ow-Yang & Maria 1963, Baker 1964, Arruda et al. 1982, Klein et al. 1990, Horosko III et al. 1997). In the 2000s, the colonisation of *Anopheles* *pseudopunctipennis*, which is considered an important vector of human *Plasmodium* spp along the Andes in several countries, was noted to have occurred by means of free intercourse (Lardeux et al. 2007). The adult mosquitoes were exposed to a blue strobe light for 20 min for several nights, encouraging them to copulate naturally under laboratory conditions. After a few generations, the researchers obtained a stable colony that reproduced by free mating. Corrêa et al. (1970) described some success in colonising and maintaining *An. darlingi *mosquitoes for about two years. Subsequently, however, Buralli and Bergo (1988) failed to achieve successful results from the same laboratory and using the same methodology. More recently, Moreno et al. (2014) described a method for *An. darlingi* colonisation that also used the strobe light approach. They reported that *An. darlingi *mosquitoes obtained after five generations were successfully infected with *P. vivax *by artificial membrane feeding similar to the previous work of Ríos-Velasquez et al. (2013) with field-captured mosquitoes.

One of the authors of this paper established colonies of two species of Neotropical anophelines 20 years ago. *An. albitarsis s.l*. was colonised in 1993 by induced copulation. After about two years of colony maintenance with induced copulation, we noticed the successful occurrence of free copulation; we used large cages with a thousand adults and a sex ratio of approximately 1:1 (Horosko III et al. 1997). *An.* *aquasalis* was settled in 1995 from the beginning by the free coupling method. In 1998, a second American malaria vector was colonised, *Anopheles albimanus*, which is one of the main vectors of malaria in Central America and in the south of Mexico (Zerpa et al. 1998). The authors used a simple and efficient maintenance method for mosquito mating and laying eggs.

Today, to the best of our knowledge and according to the specialised literature related to *Anopheles* species, only two long-term colonised American malaria vectors, *An. aquasalis *and *An. albimanus*, are maintained in laboratories and have been used for experimental studies, demonstrating that they are good models for studying the interaction of malaria vectors with *Plasmodium* species. As examples of these types of studies in *An. albimanus*, there are reports showing the susceptibility of the vector to *P. vivax* (Herrera et al. 2011, Solarte et al. 2011) and to the murine *P. berghei* (Serrano-Pinto et al. 2010, Herrera-Ortiz et al. 2011). For *An. aquasalis*, there have been studies developed by our group related to their susceptibility to *P. vivax* infection, including those related to gene expression during parasitic infection (Bahia et al. 2010 , 2011, 2013, Ríos-Velasquez et al. 2013).

## Searching for a model to study the Plasmodium interaction with an American mosquito vector

*An. aquasalis in nature: distribution, habitat and population variability* - *An. aquasalis *lives in sunny habitats with vegetation in fresh brackish water. It is believed that the mosquito prefers clean water such as that in stream pools, mangroves, ponds and ditches (Manguin et al. 1993, Grillet 2000). The demarcation of the *An. aquasalis* territory to coastal regions and its tolerance to salt water could be evolutionary adaptations that have been selected to avoid competition for food with other *Anopheles *mosquitoes (particularly during the larval phases), inserting the mosquito into the large and varied marine trophic chain (Sinka et al. 2010). The geographic distribution of *An. aquasalis* covers the southern coastal region of Central America, the Caribbean Islands and South America, but this species can penetrate eight-10 miles inland from the coast because it has a flight capacity of up to 8 km. Its presence at the Atlantic Coast has been reported from SP to Nicaragua and at the Pacific Coast from Costa Rica to Ecuador, as well as in the Antilles and Trinidad and Tobago (Faran 1980, Chadee et al. 1992, Zimmerman 1992, Consoli & Lourenço-de-Oliveira 1994).

*An. aquasalis *is an important *P. vivax *vector that is present at the Atlantic and Pacific coasts from Central America to southern Brazil. In situations in which the mosquito density increases, females can be the vectors of human malaria, especially in the absence of domestic animals, which are their usual food source. For example, Giglioli (1963) reported the effect of mechanisation on a rice farm in Guyana, which led to the disappearance of buffalo in the region. This resulted in a change in the behaviour of *An. aquasalis* that had man as its main blood source. Nevertheless, this mosquito species has been associated with several outbreaks of malaria in several countries (Deane 1986, Berti et al. 1993, Laubach et al. 2001, Mouchet et al. 2008). In most of the territory it inhabits, this species is exophilic, zoophilic and crepuscular, but in the drier northeast area it is frequently endophilic and bites human hosts. The females are opportunists, feeding in both intra and peridomiciliary areas of animals and humans. They begin to bite at sunset, reaching maximum activity in the early evening before decreasing later at night (Flores-Mendoza et al. 1996). Usually the mosquitoes rest in their peridomestic habitats before and after the blood meal.

Due to the importance of *An. aquasalis* as a vector of human malaria, it is necessary to perform studies to evaluate the genetic structure of diverse populations. In general, many *Anopheles *species are formed by complexes of cryptic species. The taxonomic elucidation of these complexes could reflect on the epidemiology and even on the control of malaria (Rosa-Freitas et al. 1998). To elucidate the dilemma of whether a given species is highly polymorphic or a complex of related species, an integrated approach of performing several studies is necessary. These studies comprise taxonomic investigations applying morphological, behavioural and molecular tools.

In its previous description, *An. aquasalis* was divided into two varieties: *An. tarsimaculatus *var.* aquacaelestis*, presenting the second hind tarsus with less than 1/6 of the length being black and *An. tarsimaculatus *var.* aquasalis*, with nearly 1/2 of its length being black (Curry 1932). Based on the morphological characters, many synonymous examples were proposed for this species. In 1941, Komp changed the name of the species known as *An. tarsimaculatus *var.* aquacaelestis* to *Anopheles (Nyssorhynchus) emilianus *by analysing egg characteristics. By studying the morphological characteristics of the eggs, larvae and adults, da Ramos (1942) renamed the same species *An. (N.) oswaldoi guarujaensis*. While working in Venezuela in 1948, Anduze (1948) found two different tonalities of mosquitoes and changed the name of the so-called *An. aquacaelestis* and *An. aquasalis* to var.* guarauno* and var.* delta,* respectively. Garcia et al. (1977) were working in Venezuela and studying several morphological characteristics in 1977 when they described *An. aquasalis* as a new species called *Ano-* *pheles (Nyssorhynchus) deltaorinoquensis*. While still working on Venezuelan mosquito populations in 1997, Maldonado et al. (1997) showed that the egg morphology of *An. aquasalis* varies within the species. More recently, a systematic study based on the morphological characteristics supported the single species status for *An. aquasalis* (Sallum et al. 2000). However, as a result of these data using morphological tools, the species complex dilemma has yet to be resolved.

To elucidate the taxonomic relationships among *An. aquasalis* and *An. emilianus* in Venezuela, Perez and Conn (1992) conducted a chromosomal banding pattern study on polytene chromosomes of different mosquito populations from endemic and non-endemic areas in that country. They observed that the banding patterns of the populations were identical to the standard chromosome map of *An. aquasalis* from Brazil. In 1993, Conn et al. analysed populations of *An. aquasalis* from Venezuela, Trinidad and Brazil using restriction enzyme digestion of mitochondrial DNA (mtDNA). The five enzymes surveyed yielded 12 mtDNA haplotypes. Estimates of mtDNA sequence divergence between all the populations were within the range of interspecific distances calculated for members of the anopheline species complexes. These results suggest a possible interspecific division in *An. aquasalis* populations north and south of the Amazon River delta (Conn et al. 1993, Linley et al. 1993). In 2002, examining variations in a fragment of the mitochondrial cytochrome oxidase I gene from five *An. aquasalis* Brazilian populations from PA and AP, Fairley et al. (2002) tested the hypothesis that the freshwater Amazon River acts as a barrier to gene flow in northeastern Brazil. Analytical results suggested that the localities within this region of northeastern Brazil constitute a single large population of *An. aquasalis* that spans the Amazon River delta.

To test the populations on either side of the Orinoco River (which is another potential freshwater barrier to gene flow for *An. aquasalis*), intragenomic heterogeneity of the internal transcribed spacer (ITS)1 and ITS2 arrays were investigated by Fairley et al. (2005) in mosquito populations from two geographic locations each in Brazil and in Venezuela and in a single location in Suriname. No sequences from either ITS had a diagnostic distribution or were informative for distinguishing between these populations, providing additional support for the status of *An. aquasalis* as a single species. In this same year, the relationship between *An. aquasalis* and other Amazonian malaria vectors was tested using the rDNA sequence ITS2. The results showed that this marker is compatible with the morphological taxonomic key established for Amazonian mosquitoes and that ITS2 sequence data has proven to be useful in species identification and potentially to solve taxonomic problems (Marrelli et al. 2005). The same results were obtained in Colombia (Cienfuegos et al. 2011). Specifically, there were only five point mutations reported for ITS2 (Fairley et al. 2005). Two interesting questions that remain are how great is the morphological and genetic variability of *An. aquasalis* in endemic areas and are these factors related to vector competence for malarial parasites.

*Experimental Plasmodium infection of mosquito vectors* - One keystone step to understanding the *Plasmodium* life cycle is the development of infectious mosquito vectors. Experimental infection models are used to understand the biology of the interaction between *Plasmodium* parasites and *Anopheles* mosquitoes. Most research projects have used laboratory models consisting of the human parasite *P. falciparum*, murine parasites *P. berghei *and* Plasmodium yoelii *and the avian parasite* P. gallinaceum* interacting with *An. gambiae*, *An. stephensi, An. albimanus *and *Ae. aegypti* mosquitoes. These mosquito species show different susceptibilities to infection by the *Plasmodium *spp. All of these parasite species are cultured in the laboratory or maintained in experimental animals, making it easy to develop experimental research, but some combinations of parasite-mosquito do not occur in nature and might not resemble the real interactions seen between parasites and their vectors (Boete 2005).

In the past, experimental infection of mosquito vectors was initiated by direct placement of the mosquitoes on the skin of malarial patients to encourage feeding (Klein et al. 1991a, c, da Silva et al. 2006b). Due to ethical issues, these types of studies are currently leaning towards the use of membrane-feeding assays instead in order to minimise the human interaction factor. Several studies have confirmed that offering a blood meal through a membrane-feeding device is as efficient as direct feeding on human skin for the study of *Plasmodium* infection of mosquito vectors. A comparative study developed by Gouagna et al. (2013) compared the field-based xenodiagnoses and direct membrane feeding assays evaluating the infectiousness to *An. gambiae* and concluded that the infection rates were similar with both methods. The membrane assay to infect mosquitoes is a simple method and can easily be applied in a laboratory without any sophisticated or complex devices.

*From P. vivax infected patients to Amazon mosquito vectors* - Today, it is possible to infect Amazon vectors in laboratories located in Manaus, the capital city of AM. The collaboration between three institutions, namely National Institute for Amazonian Research, Amazonian Oswaldo Cruz Foundation and Doctor Heitor Vieira Dourado Foundation for Tropical Medicine (FMT-HVD), has provided good conditions for developing important studies related to *Plasmodium* interaction with mosquito vectors. *P. *vivax is one of the most important causative agents of malaria in humans and is the most widespread and present parasite in America (Cruz et al. 2013 ); therefore, we decided to focus on its interaction with mosquito vectors. We used blood samples from adult volunteers (ages >18 years) infected with *P. vivax *for our experiments and diagnosed malaria using thick blood smears stained with Giemsa stain. Approximately 3 mL of blood were collected from volunteers by venipuncture. After blood collection, all the patients were treated at the FMT-HVD or in the health posts where they were diagnosed, following ethical procedures determined by the Brazilian Health Ministry.

**Fig. 4:**
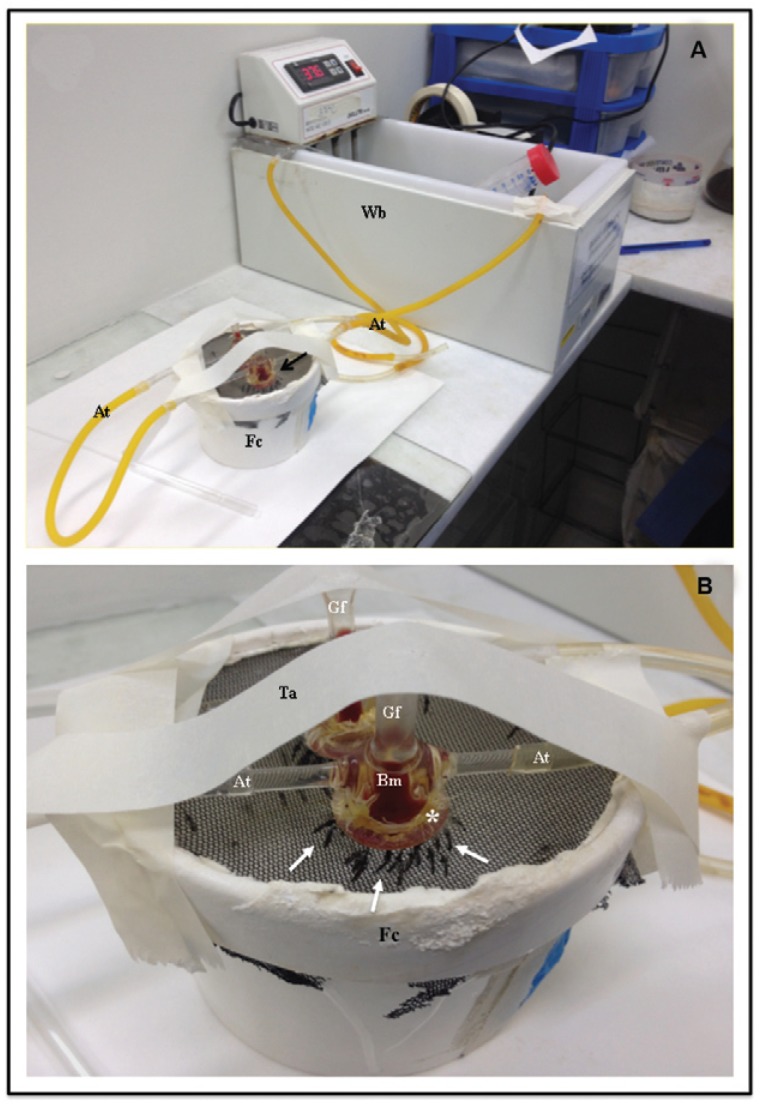
photographs of the apparatus for developing experimental infection of Anopheles aquasalis. A: a small feeding cage (Fc) for containing the mosquito is seen connected to yellow aquarium tubings (At) that are linked to a water thermal bath (Wb) with 37-39ºC circulating warm water; B: large magnification image of A in Figure showing details of the Fc. Note the glass feeder (Gf) device placed over a black mesh clothing fabric (asterisk) that is covering the Fc. The Gf is filled with an infective blood meal (Bm), linked to the At and covered by a chicken skin membrane (asterisk). Note several mosquitoes (arrows) in the feeding activity (arrows); Ta: tape for holding the Gf.

**Fig. 5:**
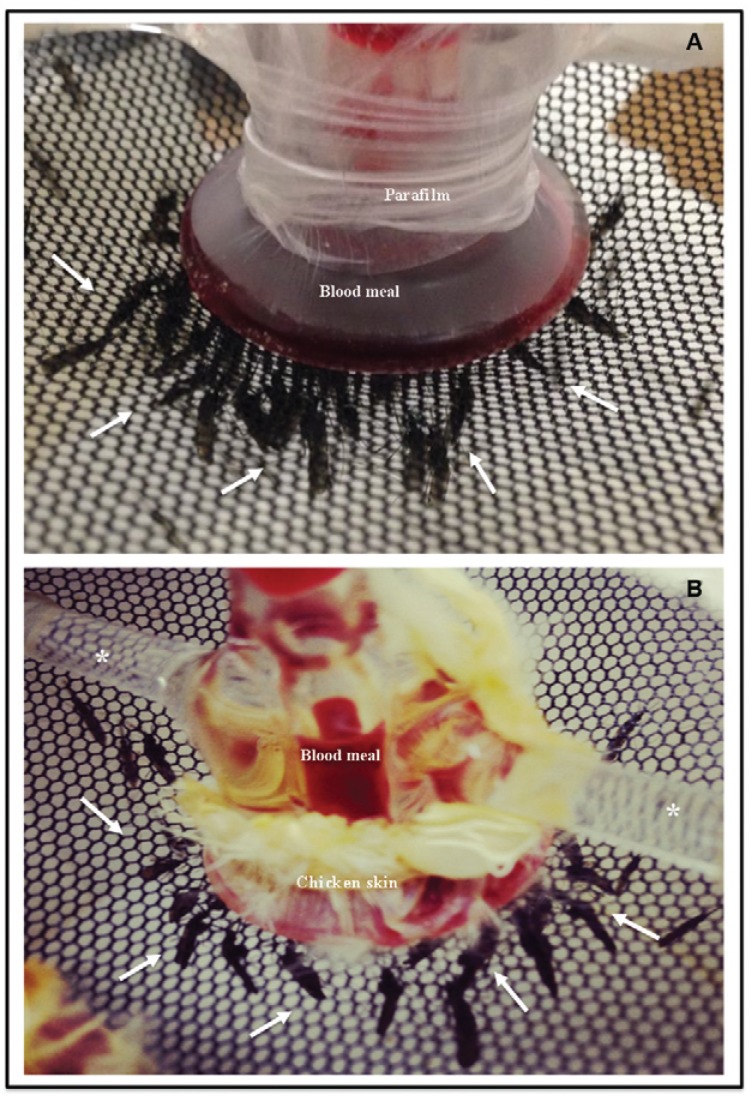
photographs showing details of the glass feeders for developing experimental infection of Anopheles aquasalis. A, B: images of the glass feeders filled with infected blood meals over black mesh clothing for retaining the mosquitoes inside the feeding cages; A: the glass feeder is covered with an artificial membrane and piece of parafilm; B: a glass feeder covered with a natural membrane, dissected chicken skin. The lateral side of the glass feeders (asterisks) are linked to aquarium tubings (not showing) for maintaining the circulating warm water. Inside the feeding cages, several mosquitoes are seen in the feeding activity (arrows in A and B).

A simple experimental protocol was used to infect the mosquito vectors (Figs 4, 5). Briefly, adult mosquitoes were sugar-starved overnight prior to infection. Blood samples infected with *P. vivax *were offered to the mosquitoes for a period of 45-90 min *via* a membrane-feeding assay through a glass feeder device (Figs 4B, 5A, B). A Parafilm^(r)^ membrane was used to cover the glass device (Fig. 5A). Other natural membranes that can also be used for the experiments include the skin from two-three day-old chicks (Figs 4B, 5B) or from young mice or hamsters. During the experimental infection, blood was held at 37-39ºC through a hose system connected to a thermal bath (Fig. 4A). Engorged mosquitoes were separated in rearing boxes. Five-eight days after ingesting infective blood meals, the midguts from the experimentally infected mosquitoes were dissected in phosphate buffered saline (PBS), stained with 2% commercial mercurochrome (Merbromin), placed under a cover glass and examined for the presence of oocysts. Additionally, 12-14 days after infection, the mosquito salivary glands were dissected in PBS in order to observe the sporozoites.

Improving the knowledge of the vectorial competence of Amazonian anopheline populations to *Plasmodium* is necessary to better understand the transmission of malaria in the region. At the end of 2013, our group published an article showing the characteristic aspects of the experimental *P. vivax *infection of key *Anopheles* species from the Brazilian Amazon and other surrounding South American countries (Ríos-Velasquez et al. 2013). This study compared the infection of four field-captured anophelines with the colonised *An. aquasalis*. The following mosquito species were studied: (i) *An. darlingi*, the major malaria vector in all countries located in the Amazon Region, (ii) *An. aquasalis* and *An. albitarsis* *s.l.*, also proven vectors, and (iii) *An. nuneztovari* *s.l.* and *An. triannulatus* *s.l.*, which have been found to be infected, but their status as vectors is not yet well defined. Larvae from the anophelines were collected in the field and reared until the adult stages, except for *An. aquasalis,* which was obtained from a well-established colony. All *Anopheles* species tested were susceptible to experimental *P. vivax* infection with the patient isolates. However, the proportion of infected mosquitoes and the infection intensity measured by oocyst number varied significantly among the species. Colonised *An. aquasalis* mosquitoes showed the highest infection intensity. It was also observed that the components of the serum (by way of inactivation) could modify the infection rates, increasing the infection in *An. darlingi* and *An. triannulatus* *s.l.*, but diminishing infection in *An. albitarsis* *s.l.* and *An. aquasalis*. The gametocyte density in the infected blood meal varied among the mosquito species. *An. albitarsis* *s.l.*, *An. aquasalis* and *An. nuneztovari* *s.l.* had higher infection rates than *An. darlingi*. This study was the first to characterise the experimental development of *P. vivax* in *Anopheles* vectors from the Amazon. The data found enabled us to infer that the *P. vivax*-vector interaction presents variations depending on the species analysed (Ríos-Velasquez et al. 2013). This fact could have a direct impact on the vector competence of the anopheline species. Moreover, this comparative study demonstrated and endorsed *An. aquasalis*, the main vector in coastal South and Central America, as a feasible laboratory model. Both *An. aquasalis*, from an established colony, and *P. vivax, *from malarial patients, are now being used by our group as a model of human malaria transmission (Bahia et al. 2010, 2011, 2013, Ríos-Velasquez et al. 2013).

*The cultivated P. falciparum parasite and mosquito vector interaction* - *P. falciparum* is the human malaria parasite with the most devastating clinical consequences. In laboratories located close to the endemic regions, it is possible to study the interaction of *P. falciparum* with mosquito vectors by feeding the mosquito with collected infected blood from local patients (Harris et al. 2012 ). However, with the introduction of the continuous culture of *P.* *falciparum,* it is now possible to study the factors involved in parasite-vector interactions in the laboratory far from the endemic areas. The first successful continuous culture was established and described by Trager and Jensen (1976).

The adaptation of several lines of *P. falciparum*-producing gametocytes in laboratories allowed the infection of colonised mosquito vectors (Trager & Jensen 1976, Carter & Miller 1979). Several studies have been performed by distinct research groups allowing the characteristics of *P. falciparum* inside some important vectors from Africa and Asia, including the molecular aspects of the interaction and the immune response to the parasite infection to be understood (Rodrigues et al. 2012 , Ramirez et al. 2014 ). Additionally, studies have shown that mosquito species exhibit a wide range of susceptibility to infection with a given *P. falciparum* line (Collins et al. 1986, Lambrechts et al. 2005 ) and different *Plasmodium* isolates also vary in their ability to infect a given mosquito strain (Niare et al. 2002, Lambrechts et al. 2005, Riehle et al. 2006 ).

A degree of adaptation was suggested between geographically isolated populations of *An. gambiae *and *P. falciparum *when an *An. gambiae* colony was successfully selected for resistance to New World *P. falciparum *isolates, but remained susceptible to those of African origin (Collins et al. 1986). Different vector-parasite interactions may have evolved through adaptation in the African *An. gambiae* and *P. falciparum,* allowing this parasite population to evade the mosquito's immune response (Lambrechts et al. 2007). African and New World *P. falciparum* populations show moderate genetic divergence (Volkman et al. 2007, Jambou et al. 2010) that could drive the differences in their infectivity. It appears that genetic differences in both the mosquito and the parasite affect the efficiency of mosquito infection and disease transmission (Molina-Cruz et al. 2012). Recent studies show that Brazilian and African lines (7G8 and NF54, respectively) infecting *An. gambiae* (African vector) differ in their ability to evade the mosquito's immune system and thioester-containing protein 1 (TEP1) (a complement like system) is correlated with parasite invasion (Molina-Cruz et al. 2012). Also of interest is an article demonstrating that *P. falciparum* development in a non-malaria vector, *Culex quinquefasciatus*, is blocked by the mosquito immune response after ookinetes have crossed the midgut epithelium and come in contact with the mosquito haemolymph (Molina-Cruz et al. 2013).

The identification of Brazilian *P. falciparum* lines that produce infective gametocytes will provide important information that will elucidate the parasite/vector interaction that is indispensable for future studies aimed at developing new strategies for blocking malaria transmission. The susceptibility of *An. aquasalis* and *An. darling*i to this parasite under laboratory conditions needs to be further investigated.

*Non-human Plasmodium species as a model for studying the interaction with mosquito vectors* - *P.* *berghei*, *P.* *yoelii* and *Plasmodium chabaudi *are murine parasites that have been adapted in the laboratory and are considered good models to investigate malaria in mammals and also to study parasite-mosquito interactions. These *Plasmodium* species have been used in different laboratories for several years to infect *An.* *gambiae, Anopheles funestus*, *An. quadrimaculatus *and *An.* *stephensi*, all of which are malaria vectors in Africa and Asia, mainly due to the vectors' high susceptibility to infection with various malaria parasite species and strains (Yoeli et al. 1964 , Vaughan et al. 1991 , Sinden et al. 2002, Alavi et al. 2003, Akaki & Dvorak 2005, Frischknecht et al. 2006, Hume et al. 2007, Lo & Coetzee 2013 , Xu et al. 2013 ).

There are several advantages of using an animal model of malaria and many research groups worldwide have begun using murine *Plasmodium-*based experimental models to better understand the interaction between malaria parasites and vectors. Essentially, these models have been helpful in the evaluation of potential interventions for malaria control and to generate and test hypotheses about the biology of human malaria and drug tests (Killick-Kendrick 1978, Jaramillo-Gutierrez et al. 2009, Xu et al. 2013).

*P. berghei* was first found in the gut and salivary glands of *Anopheles dureni* (its natural invertebrate host) in Central Africa. Later, the parasite was isolated from the vertebrate host, the tree rat, *Grammomys* *sur-* *daster, *before being was passed on to white rats and resulting eventually in the K173 strain (Vincke 1954, Yoeli 1965, Sinden et al. 2002).* P. berghei *has largely been used as a reliable experimental model for malaria studies because of its relatively simple requirements for laboratory maintenance and the availability of permanent green fluorescent-labelled strains (Franke-Fayard et al. 2004). Consequently*, P. berghei *is one of the most commonly studied *Plasmodium* species, particularly for elucidating the interactions between the parasites and their hosts (Anderson et al. 2004, Baldacci & Menard 2004, Ishino et al. 2004, Levashina 2004 , Siden-Kiamos & Louis 2004)*. P. yoelii *was originally found and isolated from rats in Central Africa. Three subspecies are recognised*, *namely* P. yoelii yoelii, P. yoelii nigeriensis *and *P. yoelii killicki, *and they are widely used to study host immune responses and the genetic basis of parasite phenotypes.* P. chabaudi* is a parasite of the African thicket rat, *Thamnomys rutilans*; it has been adapted to develop in the laboratory mouse and is one of the best laboratory models for the study of malaria. The species is one of the most common murine models that have been utilised within vaccine research. *P. berghei *and *P. yoelii* transgenic lines that constitutively express green fluorescent protein (GFP) can develop throughout the entire life cycle in the vertebrate host and these mosquito vectors have been very useful in laboratorial experiments.

*P. gallinaceum* is an avian malaria parasite that is phylogenetically closer to *P. falciparum* than it is to many other malaria species (McCutchan et al. 1996, Roy & Irimia 2008) and has intriguingly become very useful in laboratories because it can be infected and complete its entire cycle in *Ae. aegypti *mosquitoes and in* Aedes fluviatilis *(Tason & Krettli 1978, de Camargo et al. 1983, Pimenta et al. 1994, Gupta et al. 2005). This model is now widely used for understanding the cell biology of parasitic infection and the routine chemotherapy test in chicks (Carvalho et al. 1992, Rocha et al. 1993a, b, Ramirez et al. 1995, Krettli et al. 2001, da Rocha et al. 2004, Maciel et al. 2008, Rodrigues et al. 2008).

Few studies regarding New World vectors have been developed to date.* An. albimanus, *a Central America malaria vector, can be infected by* P. yoelii, *but cannot be effectively infected by* P. berghei *(Vaughan et al. 1994, Noden et al. 1995, Brucker & Bordenstein 2013). However, Frischknecht et al. (2006) demonstrated that a transformed GFP-*P. berghei* line can complete its life cycle in this North American vector. However, the susceptibility of two important human malaria vectors of this parasite in South America,* An. aquasalis *and* An. darlingi, *requires further investigation under laboratory conditions. It was recently shown that* An. funestus, *an important vector in Sub-Saharan Africa, is permissive for* P. berghei *development, which is in contrast with previous reports (Xu et al. 2013). This kind of work highlights the importance of fully testing New World anopheline species for *P. berghei *experimental infections using different parasite strains and mosquito populations.

The establishment of experimental infections using* An. aquasalis *mosquitoes from colonies and* P. yoelii *and* P. berghei *parasites could provide an interesting model for studying malaria in the Amazonian scenario. It could definitely be the first step in finally understanding the biology underlying* P. vivax *and/or* P. falciparum *infection of Brazilian vectors.

## The immune response of the mosquito vector to Plasmodium infection

Understanding the molecular mechanisms involved in the development of the parasites in the vectors is an important step in determining the interaction process and vectorial competence. Mosquitoes, like other organisms, produce humoral and cellular immune responses. A large range of molecules can be produced against pathogens such as bacteria, fungi, viruses and *Plasmodium* spp and can be secreted by mosquito organs and tissues as fat bodies, haemocytes and midgut cells (Yagi et al. 2004, Cirimotich et al. 2010 ). Recent studies using microarrays and transcriptome techniques have described how *Plasmodium *parasites can modulate the expression of immune genes in *An. gambiae* and *An. stephensi* (Dimopoulos et al. 2002, Xu et al. 2005, Dong et al. 2006 , Baton et al. 2009). Actually, many studies have produced evidence supporting the fact that the vectorial competence of a determined vector depends on the action of the mosquito immune system during the infection process with *Plasmodium *species.

During several steps of the life cycle, mosquito immune defences can kill parasites, thereby controlling or eliminating the infection. Once *Plasmodium* parasites are ingested by female mosquitoes during blood feeding, they face the harsh environment of the digestive tract. It has been previously observed that these parasites can negatively or positively modulate the gene expression and activity of many of the mosquito's digestive enzymes (Gass & Yeates 1979, Jahan et al. 1999, Somboon & Prapanthadara 2002). There are several phenomena related to the mosquito vector's defences that can occur. For example, the production of nitric oxide synthase (NOS) by the vector occurs from the period before the invasion of the intestinal epithelium to the time when the parasite crosses the epithelial cells. NOS is responsible for activation of the production of the antimicrobial peptides that are responsible for the death of a large number of ookinetes in the insect gut (Luckhart et al. 1998, Dimopoulos et al. 2001, Olayan et al. 2002, Herrera-Ortiz et al. 2011). Moreover, NOS is also an important component of the nitration process in *Plasmodium*-invaded midgut cells and targets parasites for complement activation through TEP1 protein (Oliveira et al. 2011). Additionally, due to this immune response (at least for the human *Plasmodium*), less than 10 ookinetes can successfully cross the intestinal epithelium and form viable oocysts (Ghosh et al. 2000). This means that only a small proportion of the ingested parasites will be able to successfully escape the interior of the intestine, cross over the PM and invade the epithelial cells of the intestine. Activation of the melanisation cascade may also occur during the crossing of the intestinal epithelium. A cascade of serine proteases which activates PPOs through a second cascade leads to the deposition of melanin and free radicals that are involved in the death of ookinetes (Luckhart et al. 1998, Hoffmann et al. 1999, Ghosh et al. 2000, Ligoxygakis et al. 2002, Cirimotich et al. 2010). The ookinetes that survive the onslaught of the immune system will release the sporozoites. In the haemolymph, the phagocytosis of sporozoites by mosquito haemocytes has been described in *Ae. aegypti *and *An. gambiae* (Hillyer et al. 2003, 2007). In addition to their phagocytic activity, these haemocytes are able to secrete substances that assist in promoting the death of the parasite (Blandin & Levashina 2007 ). Antimicrobial peptides that are rapidly produced by the fat body of the insect also represent an important step in fighting the infection. Actually, there is an intensive role that the mosquito's immune system has to constantly undergo in order to fight back the infection.

The insect's defense mechanisms are activated by intracellular immune signalling pathways. Toll, immunodeficiency (IMD) and JAK/STAT are the three major immune pathways, first described in *Drosophila* and then in *Anopheles* (Cirimotich et al. 2010). The Toll pathway activation by *P. berghei* is able to restrain parasite survival in *An. gambiae* (Frolet et al. 2006). Over-activation of this pathway by silencing the negative regulator cactus dramatically reduced *P. berghei *loads in *An. gambiae*, *An. stephensi *and *An. albimanus*, but not *P. falciparum *numbers in these same mosquito species (Garver et al. 2009). Interestingly, the IMD pathway plays an important role in limiting *P. falciparum *infection. Depletion of caspar, the negative regulator of the IMD pathway, promotes a *P. falciparum*-refractoriness phenotype in *An. gambiae *mosquitoes. However, the same phenotype is not achieved when *P. berghei *is used (Garver et al. 2009).

In *An. gambiae*, the JAK/STAT pathway mediates the killing of *P. falciparum *and *P. berghei *in the late infection phases after midgut invasion. Disruption of this pathway by silencing the transcription activator, STAT-A, promotes *P. berghei *oocyst development. Meanwhile, the over-activation of the JAK/STAT pathway by depletion of the suppressors of cytokine signalling triggers NOS expression and decreases the infection levels (Gupta et al. 2009).

Reactive oxygen species (ROS) are generated by mitochondrial activity and/or activation of the immune system in mosquitoes (Kumar et al. 2003, Molina-Cruz et al. 2008, Gonçalves et al. 2012). In *An. gambiae*, the ROS-producing dual oxidase protein and an haemeperoxidase (HPX2) are able to secrete a dityrosine network. This network prevents strong immune activation of the midgut by commensal gut bacteria. When *Plasmodium* ookinetes invade epithelial cells, the dityrosine network is disrupted and a high level of NO, which has a strong negative effect on parasite survival, is produced (Kumar et al. 2010). In addition, the invasion of the *An. gambiae *midgut epithelium by the *P. berghei *ookinetes induces the expression of a nicotinamide adenine dinucleotide phosphate (NADPH) oxidase, NADPH oxidase 5 and HPX2, which catalyses protein nitration leading to parasite opsonisation and killing through complement action in the mosquito's haemolymph (Oliveira et al. 2011). Although ROS can promote parasite killing, they can also be hazardous to mosquito cells. Therefore, ROS production should be compartmentalised and their life-span must undergo fine regulation by the activation of detoxifying enzymes such as catalase and superoxide dismutase (SOD). In *An. gambiae*, catalase expression and activity is inhibited by *P. berghei *infection. The silencing of this enzyme decreases *P. berghei *survival (Molina-Cruz et al. 2008), emphasising that ROS are important immune effectors against *Plasmodium *parasites.

Another major process in insect defense is the melanisation immune response that is present in the major classes of arthropods. Factors present in the haemolymph mediate melanin synthesis when the recognition of non-self is activated and a CLIP cascade culminates in the limited proteolysis and conversion of inactive prophenoloxidase proenzyme (PPO) into active phenoloxidase (PO). Subsequent oxidation of phenols by PO leads to the production of quinones that polymerise to form melanin. Several serine proteases have been identified and characterised in the haemolymph of *Anopheles *in the presence of *Plasmodium*. Changes in the conformation of some membrane receptors activate a serine protease, which in turn triggers the activation of the PPO cascade that activates the melanisation immune response. PO is a very active enzyme and its activation intermediates are toxic both to invading microorganisms and for the insect itself. Therefore, its activation is limited to the site of infection and if not, it could lead to widespread and lethal melanisation for insects. In the plasma and haemocytes, inhibitory proteins such as serpins (SRPNs) can be found that regulate the activity of serine proteases (Volz et al. 2006). In mosquitoes, SRPNs regulate the cascade of PPO and determine whether or not malaria parasites are lysed, mainly *via* the activation of the Toll and IMD pathways (Gulley et al. 2013).

Many functional genetic studies have demonstrated in the *An. gambiae*/*P. berghei* system that melanisation can eliminate dead ookinetes (Blandin et al. 2004) or directly mediate ookinete killing, based on the mosquito's genetic background (Volz et al. 2006). The melanisation response of *Plasmodium *has been particularly followed in refractory mosquitoes such as the *An. gambiae *strain (L35), which melanises most *Plasmodium *species including the Brazilian *P. falciparum *7G8 line; it is highly susceptible to some African *P. falciparum *strains such as LE5 and NF54 (Collins et al. 1986). Recently, Molina-Cruz et al. (2013) investigated whether these parasite lines differed in their ability to evade the mosquito's immune system. Silencing key components of the mosquito's complement system (TEP1, LRIM1 or APL1) prevented melanisation of 7G8 parasites, reverting to the refractory phenotype. In contrast, it had no effect on the intensity of the infection with NF54, indicating that this line is able to evade the mosquito's complement system. Furthermore, when L35 females were co-infected with a line that is melanised (7G8) and one that survives (3D7), this resulted in mixed infections with both live and encapsulated parasites in individual midguts. The African 3D7 parasites were able to evade the mosquito complement system even when 7G8 parasites were being melanised, indicating that immune evasion is parasite-specific and not systemic in nature. These findings suggest that evasion of the *An. gambiae *immune system by *P. falciparum *may be a result of parasite adaptation to sympatric mosquito vectors and may be an important factor driving malaria transmission (Molina-Cruz et al. 2012).

In the interaction studies of *Plasmodium *with their vector, more attention has been paid to the TEP1 that has a similar structure to that of vertebrate C3. Mosquito haemocytes synthesise and release TEP1 in the haemocoel. TEP1 acts as an opsonin, promoting the phagocytosis of Gram-negative and Gram-positive bacteria in a thioester-dependent manner (Levashina et al. 2001). It was also observed that TEP1 can bind and mediate the killing of the midgut stages of *P. berghei *parasites (Blandin et al. 2004) and efficient binding of TEP1 to the ookinetes requires previous parasite targeting by midgut protein nitration (Oliveira et al. 2011). Specifically, TEP1 binds to the surface of the *P. berghei *ookinetes escaping from the basal side of the mosquito midgut epithelium, mediating the death of the parasite (Blandin et al. 2004). Moreover, TEP1-depleted susceptible and refractory (L35) *An. gambiae *mosquitoes showed enhanced development of *Plasmodium *oocysts, clearly demonstrating its anti-parasitic effect (Blandin et al. 2004) for *P. berghei *(Molina-Cruz et al. 2012) and for *P. falciparum*. Considering the LRIM1, LRR and APL1C cited in the above paragraph that also displayed a similar knock-down phenotype to that of TEP1 and increased *P. berghei *oocyst numbers in susceptible and L35 refractory mosquitoes, as well as inhibiting ookinete melanisation (Osta et al. 2004 , Riehle et al. 2008, Povelones et al. 2009), there is a functional collaboration between these three proteins in mosquito anti-parasitic defence. Further studies of these complex molecules are necessary for a complete understanding of the innate immunity of these malarial vectors.

Haemocytes are the main players of the insect cellular response. The haemocyte types can vary greatly from flies to mosquitoes (Blandin & Levashina 2007). In *An. gambiae*, the main haemocyte populations are prohaemocytes, progenitor cells, granulocytes, phagocytic cells and oenocytoids (Rodrigues et al. 2010). They are responsible for the melanisation and encapsulation of pathogens in the haemolymph. In addition, haemocytes can also produce humoral effectors that target *Plasmodium *parasites (Pinto et al. 2009). Recent studies have demonstrated that different *Plasmodium *species can trigger haemocyte differentiation in* An. gambiae *(Ramirez et al. 2014) and an increase in the granulocyte population is associated with immune protection towards subsequent *P. berghei *infections (Rodrigues et al. 2010).

The *Plasmodium* life cycle is a complex process and one could argue that this complexity is due to the parasite's ability to alter itself on a cellular and molecular level. Recent studies have determined that the expression of *Plasmodium *surface proteins can control the vector infection. The *P. falciparum *gamete surface protein genes *Pfs48/45 *and *Pfs47 *have been shown to have highly polymorphic regions (Conway et al. 2001, Anthony et al. 2007). Population studies have demonstrated an extreme geographical divergence of allele frequencies for both the *Pfs48/45 *and *Pfs47 *genes. This strong population structure is not observed in other *P. falciparum *genes. The *Pfs48/45 *and *Pfs47 *genes have seven and 18 single nucleotide polymorphisms (SNPs), respectively, while other genes have fewer SNPs. The African lines had the most diverse combinations of these genes, whereas parasites from Brazil and Peru have the same SNP combination. Recently, Molina-Cruz et al. (2013) identified *Pfs47 *as an essential survival factor for *P. falciparum *that allows the parasite to evade the immune system of *An. gambiae*. *Pfs47 *suppresses midgut nitration responses that are critical in activating the complement-like system. Thus, the disruption of *Pfs47 *reduced parasite survival in the mosquito. These authors also provide evidence that *Pfs47 *population structure may be due to the adaptation of *P. falciparum *to different *Anopheles *vector species present outside of Africa. Understanding the molecular mechanisms involved in this step is crucial to interfering with the development of *Plasmodium *in mosquitoes.

## Immune response of An. aquasalis to P. vivax infection

Because the genome sequence of this mosquito is still not available, differential subtraction mRNA libraries were generated to investigate how *P. vivax* infection modulates *An. aquasalis* gene expression (Bahia et al. 2010). Infection down-regulated the expression of the genes related to mosquito embryogenesis and energy metabolism, which was consistent with the notion that the activation of the immune system towards *Plasmodium* has a negative impact on reproductive fitness (Hopwood et al. 2001, Ahmed & Hurd 2006). In contrast, only 3% of the obtained sequences were related to immunity. This weak immune activation could be associated with a high compatibility between *P. vivax* and *An. aquasalis,* as demonstrated for other parasite-vector combinations (Jaramillo-Gutierrez et al. 2009).

Regarding the harsh environment of blood digestion in the *P. vivax*-*An. aquasalis *model, the expression of a chymotrypsin-like protease was heavily inhibited by infection 24 h after this infection occurred, showing that the parasite can negatively modulate this gene expression. The same effect was not observed for a carboxypeptidase A-like protein also found in this anopheline (Bahia et al. 2010). However, *P. vivax* infection induced the expression of a member of the SRPN family. These are classical inhibitors of serine proteases that participate in blood digestion and the melanisation cascade (Dana et al. 2005, Michel et al. 2005 ). It is still unclear, however, whether these changes in digestive enzymes could have a protective effect on *P. vivax* development in *An. aquasalis* mosquitoes.

In *P. vivax*-infected *An. aquasalis*, catalase and SOD expression was induced 36 h post-infection (p.i.) in the whole mosquitoes. This induction was not observed in the infected midguts. However, midgut catalase and SOD activities were significantly lower 24 h after infection, indicating that *P. vivax* parasites can modulate the detoxifying response post-transcriptionally (Bahia et al. 2013). The silencing of catalase increased *P. vivax* infection and prevalence. These results are in contrast with previous reports for *An. gambiae* (Molina-Cruz et al. 2008) and suggest that ROS are necessary for *P. vivax* development in *An. aquasalis *mosquitoes, leading this parasite to manipulate the detoxification system accordingly.

The role of IMD and Toll pathways on the *P. vivax*-*An. aquasalis* interaction remains unclear. *P. vivax* can induce the expression of the antimicrobial peptide cecropin in *An. aquasalis* mosquitoes (Bahia et al. 2010) and cecropin production is under the control of IMD and Toll pathways in other mosquito species (Meister et al. 2005 , Moon et al. 2011, Pan et al. 2012 ).

Bahia et al. (2011) showed that the JAK/STAT pathway is also activated in *P. vivax*-infected *An. aquasalis* mosquitoes, but at an earlier stage than previously reported for *An. gambiae* (Gupta et al. 2009). The expression of STAT, the negative regulator protein inhibitor of activated STAT1 and the immune effector NOS was induced by *Plasmodium* at 24 and 36 h p.i. NOS is an important component of the nitration process that targets parasites for complement activation (Gonçalves et al. 2012). Besides to silencing of STAT promoted *P. vivax *development in *An. aquasalis* mosquitoes. The effect of the STAT pathway on *P. vivax* infection at later stages is yet to be investigated.

## Consideration of anopheline genomes and those of New World vectors

The 2002 publication of the *An. gambiae sensu stricto* (Holt et al. 2002) and the *P. falciparum* (Gardner et al. 2002) genomes marked a breaking point in the field of malaria vector biology research. The *Anopheles* project wrapped together decades of classic genetics knowledge, allowing us to better understand issues such as chromosome and gene architecture. It also allowed vector biologists to plunge into the area of comparative genomics through which the first comparisons made (Christophides et al. 2002, Zdobnov et al. 2002) addressed matters such as the composition of the immunity-related gene repertoire. In the post-genome era, several genetic engineering tools and strategies for vector control have arisen, have been implemented and have been assessed (Alphey et al. 2002, Lycett & Kafatos 2002, Scott et al. 2002, Benedict & Robinson 2003, Riehle et al. 2003, Tabachnick 2003, Toure et al. 2004, Sinkins & Gould 2006, Takken & Knols 2009, Isaacs et al. 2011, Sumitani et al. 2013). Nevertheless, the high diversity and plasticity that *Plasmodium* parasites have shown in vertebrate and invertebrate hosts have led to the assumption that the parasites evolve faster and adapt rapidly, more so than human and anopheline hosts (Carius et al. 2001, Cohuet et al. 2010 ). As a consequence of this phenomenon and with the experiences thus far accumulated, the vector biology community understood that sequencing the genomes of multiple mosquito and parasite species would be imperative to understanding and manipulating the vector-parasite interactions.

For this purpose, efforts were jointly channelled *via* the *Anopheles* Genomes Cluster (AGC), which in 2008 formed the basis of what would become the first anopheline comparative genomics consortium (Besansky 2014). The committee identified and selected 16 mosquito species whose genomes and transcriptomes were about to be published (Neafsey et al. 2013) and made available through the VectorBase (Megy et al. 2012). Unfortunately, *An. albimanus* is the only American vector listed in the project and no attention was paid to the Amazon mosquitoes that are the vectors of the majority of the human cases on the continent.

The evolutionary vector-parasite dynamics, vectorial competence traits and mosquito behaviour could have been shaped by multiple factors such as specific genotype combinations. Experimental evidence and theories explaining how the genomic composition of a mosquito species determines whether it is refractory or susceptible towards infection by a species (strains) of *Plasmodium *parasite have been published (Billingsley & Sinden 1997, Norris et al. 2001, Osta et al. 2004, Lambrechts et al. 2005, Riehle et al. 2007, Jaramillo-Gutierrez et al. 2009, Harris et al. 2010). There is also a great body of literature connecting vector biology with non-genetic components such as ecological factors (Schmid-Hempel & Ebert 2003, Lambrechts et al. 2005, Tripet et al. 2008 , Tripet 2009, Wolinska & King 2009).

As stated by the AGC (Moreno et al. 2010, Besansky 2014), sequencing the genome of mosquito species that capture and represent the evolutionary and phenotypic divergence within the anopheline vectors distributed throughout the world is critical. It is the consensus among the community that envisioning a eukaryote genome project requires looking at it as a continuous process of innovation, re-sequencing and annotation (Li et al. 2006 , 2010, Sharakhova et al. 2007, Moreno et al. 2010). Together with the *An. gambiae* *s.s.* genome, other annotated anopheline assemblies will provide a platform for gaining genome-wide evolutionary and population genetic insights into the mechanisms of speciation and the biological processes that influence the ability of the mosquitoes to transmit malaria parasites to humans.

It has also been brought to the attention of the vector community that the genomic aspects of vectorial capacity and competence have not been uniformly studied (Cohuet et al. 2006, 2010) and some have been largely overlooked, both in terms of the species analysed and the gene families addressed by experimental biology. For example, rapid progression has been made regarding mosquito immunity, insecticide resistance and olfaction genetics. However, the genetic determinants of parasite virulence, mosquito adaptation to human environments and the evolutionary forces exerted on vectors by the parasite and the microbiome associated with them, are still progressing slowly. The area of comparative genomics is rapidly evolving and developing tools. Therefore, the number of questions that vector biology can answer through sequenced and published genomes has expanded (Zdobnov et al. 2002, Reddy et al. 2012). Major analytical themes now include topics such as molecular evolution and speciation, chemoreception, circadian rhythm, development, repetitive and transposable elements, reproduction, secretomes, rearrangements of chromosomal architectures, neuropeptides and behaviour, blood/sugar metabolism and so on.

The Neotropical vectors represent an interesting target to understand how competent malaria transmission evolved in a different ecological setting and also followed different human settling conditions (Fagundes et al. 2008, Hubbe et al. 2010, O'Rourke & Raff 2010, Bodner et al. 2012, Yalcindag et al. 2012). It is believed that the interactions between the actors of the malaria transmission triad - humans, Neotropical vectors and *Plasmodium* parasites - are relatively recent on the American continent. For example, the main Neotropical malaria vector, *An. (Nyssorhynchus) darlingi*, which diverged from *An. (Cellia) gambiae* approximately 100 million years ago, could have evolved in a human and parasite-free environment for several million years (Moreno et al. 2010).

When we add up all of the biological evidence and take into account the fact that malaria is a malady that still imposes a high burden upon the people who live in the Amazon Basin (> 500 thousand cases are reported annually), sequencing the genome of a Neotropical vector seems important. Thus, in 2013, this became a reality with the publication and upload onto the VectorBase of the *An. darlingi* genome (Marinotti et al. 2013). This project was performed at the behest of the Brazilian National Council for Research and set a cornerstone for future basic and applied comparative genomics studies. Such research endeavours will be able to start answering long sought-after answers regarding the biology of malaria in an American context and will focus on generating genetic and chemical tools (e.g., insecticides, bacterial larvicides and paratransgenesis strategies) for vector control that better adjust to the ecological and public health conditions in Latin America.

The Brazilian malaria research network is aware of the pitfalls that were addressed and elegantly presented by the AGC regarding the ordeals of the *de novo* assembly of complex eukaryote genomes. Critical aspects of genome sequencing and assembly have been proposed for discussion in the vector biology community due to the open nature of the AGC work. Such topics include: the necessity of isogenic colonies, DNA template quality, genomic library building techniques and heterozygosity-solving algorithms, amongst others. It is the opinion of the Brazilian malaria research network that the time is right to embark on the establishment of a suitable model for research that benefits from the experience and data generated by the *An. darling*i genome and together expands and enriches the depth of knowledge of American vector biology.

As proof of the steps being taken by research groups in Brazil towards the advancement of genomic sciences, we can also mention the ongoing *An. aquasalis* genome project. This will bridge the vacuum that currently exists between the *An. darlingi* model and its use in experimental biology research. The absence of colonies of this species in several laboratories and the highly heterozygous nature of its genome assembly still hinder its potential as a research model.

The *An. aquasalis* species has viable, operating colonies throughout Brazil. It is pertinent that *An. aquasalis* has been used in experimental infections and transmission assays with multiple *Plasmodium *species. Therefore, this species is positioned as a top model for the understanding of malaria transmission within the Brazilian context. The peculiar bionomics of the *An. aquasalis* mosquito (Sinka et al. 2010) has prompted us to expand and explore other "genomic" areas, in particular the reconstruction of the associated consortium of bacteria and viruses that could be predicted from the massive parallel sequencing process is of interest. Next Generation Sequencing (NGS) technology has evolved into an impressive tool that ranges from genome assembly to microbiome screening (Mardis 2011). When carefully implemented and combined with the experimental designs of genome sequencing projects, metagenomics could become a key element to deconvolute the complex inner insect ecosystem.

As a final thought, we believe that tailored measures of vector control that respond to local conditions and transmission patterns are sorely needed in our region. Targeted interventions based on the growing existence of genomic data pertaining to tandems of Neotropical vectors and *Plasmodium* parasites could enhance the control strategies that already exist. Building the capacity to generate and use comparative genomics data from local anopheline species is therefore justified.

## Modulation of Plasmodium infection by the mosquito vector microbiota

Amongst the metazoans, insects are by far the most diverse and abundant clade (Basset et al. 2012). Their success can be explained in part by the relationships they have established with beneficial members of their associated microbiome. The term microbiota defines the microbial communities that stably or transiently colonise insect epithelia as well as intracellular compartments and target organs. They may vary from bacteria to viruses, yeasts and protists. The bacterial component of this ecosystem is to date the most studied and characterised (Ng et al. 2011 a, Gendrin & Christophides 2013, Minard et al. 2013). These symbiotic microbiomes or consortiums are beneficial for their insect hosts in many ways (Dillon & Dillon 2004, Azambuja et al. 2005, Thomas et al. 2012, Engel & Moran 2013 ), including the following: as dietary supplementation, for the enhancement of digestive mechanisms, to help tolerate environmental perturbations, for protection from parasites (Degnan & Moran 2008) and pathogens (Nartey et al. 2013) and for the maintenance and/or enhancement of host immune system homeostasis. Furthermore, the absence or elimination of the microbial fauna and even the modification of its composition can reduce the fitness of the harbouring insect (Thomas et al. 2012). This observed influence of the microbiome on its host has been referred to as the extended phenotype and can range from mutualism to parasitism, as well.

Recently, the study of microorganisms living in the insect gut has increased considerably. The last decade has seen the publication of multiple relevant studies ranging from diversity screening metagenomic surveys (Baumann 2005, Lindh et al. 2005 , Carpi et al. 2011, Dinparast et al. 2011, Lindh & Lehane 2011, Ng et al. 2011a, b, Chavshin et al. 2012) to molecular studies on how the gut bacteria interact with the host's immune system and respond to infection (Azambuja et al. 2005, Chouaia et al. 2010, Boissiere et al. 2012).

It is not within the scope of this review to provide an exhaustive analysis on metagenomics or the architecture and dynamics of this micro-ecosystem within Culicine vectors. Recent revisions cover these topics substantially and creatively (Dillon & Dillon 2004, Engel & Moran 2013, Gendrin & Christophides 2013, Minard et al. 2013). Our aim is to briefly call attention to recent advancements that malaria vector control has generated regarding microbiota and its association with vector competence traits. Many of them have been greatly enhanced by the use of metagenomic tools that have allowed us to discover and explore how microbial species could be used in paratransgenesis and malaria transmission-blocking strategies.

Metagenomics emerged as a derivation of classic microbial genomics with the key difference being that it bypasses the requirement for obtaining pure cultures for sequencing (Glass et al. 2010, Huttenhower 2012, Kim et al. 2013). We now have the ability to obtain genomic information directly from microbial communities in their natural habitats and study them in a concerted manner, describing their species composition and even predicting the potential genomic functions and metabolic capabilities they possess (Wooley et al. 2010, Williamson & Yooseph 2012).

As NGS has skyrocketed, our potential to generate genomic data benchmarking (Ansorge 2009) has gained relevance, providing guidance to experimental biologists that encounter themselves with a myriad of available bioinformatics tools (Delcher et al. 2007, Huson et al. 2007, Meyer et al. 2008 , Angly et al. 2009, Clemente et al. 2010, Glass et al. 2010, Gerlach & Stoye 2011, Jiang et al. 2012). As users of such technology, we would like to stress that when designing experiments that encompass metagenomic data generation, it is imperative to consider points such as: sampling techniques, DNA/RNA extraction protocols, sequencing platforms, assembly, taxonomic binning, gene annotation tools, statistical analysis and data/meta-data sharing formats (Wommack et al. 2008, Tanenbaum et al. 2010, Wooley et al. 2010, Thomas et al. 2012). The availability of standardised procedures (Field et al. 2008, Tanenbaum et al. 2010) and platforms for data storage and sharing are becoming increasingly important to ensure that the output of individual projects can be assessed and compared (Thomas et al. 2012).

Metagenomic screening assays are now being used to determine the diversity of microorganisms and viruses residing in arthropod vectors of medical importance. Such assays allow human health agencies and research groups to monitor endemic infections, perform real-time surveillance of newly emerging zoonotic pathogens, discover etiological agents and discover how they associate with and within their host (Bishop-Lilly et al. 2010, Carpi et al. 2011, Ng et al. 2011a, b, Mokili et al. 2012).

Due to their importance as vectors of malaria, anopheline mosquitoes have been the targets of multiple efforts to profile their microbiota (Gendrin & Christophides 2013). Behind this effort lies the knowledge that bacteria living in the midgut have been found to modulate the response of the mosquitoes towards *Plasmodium* infection (Pumpuni et al. 1993 , Dong et al. 2009, Boissiere et al. 2012, Eappen et al. 2013), have the potential to block infections and can be used as genetic transformation vehicles (Pumpuni et al. 1993, Dong et al. 2009, Weiss & Aksoy 2011, Boissiere et al. 2012, Ricci et al. 2012, Eappen et al. 2013). Below, we summarise some of the key findings regarding the impact of microbiota on the *Plasmodium*-Culicidae interaction model.

Both laboratory and field mosquito strains have been found to be associated with microbial organisms that particularly colonise the gut. They consist primarily of Gram-negative bacteria of the Enterobacteriaceae family. Field populations of *An. gambiae* and *An. funestus* were found to contain 16 bacterial species spanning 14 genera (Lindh et al. 2005). The laboratory populations of *An. gambiae* and *An. stephensi* also presented a wide variety of bacteria, especially of the genus *Asaia*, *Enterobacter*, *Mycobacterium*,* Sphingomonas*,* Serratia* and *Chryseobacterium* (Favia et al. 2007, Dong et al. 2009). Bacteria of the *Asaia* genus were also found in *Ae. aegypti* mosquitoes (Pidiyar et al. 2004, Rani et al. 2009, Gaio et al. 2011). In addition, beyond the digestive tract, studies have shown that the species of this genus are also able to colonise the salivary gland and ovaries of mosquitoes and are usually acquired through vertical transmission (Favia et al. 2007).

It has been shown that gut bacteria may have an impact on vectorial competence by inhibiting the sporogonic development of malaria parasites within the mosquito vector (Pumpuni et al. 1993, 1996, González-Cerón et al. 2003, Dong et al. 2009, Cirimotich et al. 2011). Pumpuni et al. (1993, 1996) also showed, whilst manipulating the bacterial content, that Gram-negative bacteria inhibit oocyst formation in whole or in part and that the same action was not observed with Gram-positive bacteria.

Evidence of this influence of the intestinal microbiota on the life cycle of the parasites has been demonstrated for other insects such as sandflies and tsetse flies (Schlein et al. 1985, Welburn & Maudlin 1999).

Recent studies suggest that *Enterobacter* species in the gut of *Anopheles arabiensis *mosquitoes originating from Zambia act directly on *P. falciparum,* blocking the development of the parasite and making this population refractory to infection. This refractoriness was associated with the generation of the ROS that interfere with the development of the parasite and kills it before its invasion of the intestinal epithelium (Cirimotich et al. 2011).

Previous studies suggest that bacteria in the gut lumen modify the intestinal environment and inhibit the development of parasites by the actions of the immune system by overexpression of immunity genes, culminating in an increased rate of production of antimicrobial peptides (Pumpuni et al. 1996, Ratcliffe & Whitten 2004, Michel & Kafatos 2005). Such peptides are likely to play a key role not only in the control of pathogenic or symbiotic bacteria, but also in the development of infections by parasites (Beard et al. 2001, Boulanger et al. 2004). Interestingly, the mosquito immune system acts against bacterial growth and also eliminates a large number of parasites modulating the intensity of infection in mosquitoes infected with *P. berghei* or *P. falciparum* (Meister et al. 2009).

It has also been proposed that certain bacteria taxa can induce a reductive environment within the mosquito midgut, thus aiding in the detoxification of reactive oxygen and nitrogen species, a fact that would allow for an aggressive immune response of the mosquito when infected by the parasite (Wang et al. 2011).

In *Ae. aegypti, *antibiotic treatment affects the progression of *P. gallinaceum *infection. It was observed that mosquitoes treated with kanamycin partially inhibited the sporogonic development of *P. gallinaceum,* while carbenicillin-treated mosquitoes were significantly more susceptible to infection. Although both antibiotics are effective against Gran-negative bacteria, carbenicillin also affects Gram-positive bacteria (AS Orfano et al., unpublished observations).

Recent results obtained in our laboratory show that the expression of AMPs of *Ae. aegypti* mosquitoes is modified with antibiotic treatment and subsequent infection with *P. gallinaceum*. Insects treated with kanamycin had increased expression of defensin 24 h and 36 h after being fed an infective blood meal, in comparison with a group of mosquitoes not treated with antibiotics that were fed an infective blood meal. This period in particular occurs when the ookinete begins to invade the intestinal epithelium, reducing infection. Similar results were observed in similar experiments with *An. gambiae* mosquitoes upon infection with *P. berghei*; a peak of defensin expression was detected at 26 h after the antibiotic-treated mosquitoes were fed an infective blood meal (Richman et al. 1997). In our model, when the insects are treated with carbenicillin and infected, the expression levels of defensin were inferior to those of the control mosquitoes at 24 h and 36 h after blood feeding, revealing a less active immune system, which probably leads to a greater susceptibility to the avian malaria parasite.

In conclusion, we would like to highlight the fact that vector biology has made great advancements over the past years and many results have been attained by "synergic" approaches with computational science as a key element. Many interesting theories are now being discussed and explored regarding the hologenomic basis of speciation (Nikoh et al. 2008, Rosenberg & Zilber-Rosenberg 2011, Ni et al. 2012 , Brucker & Bordenstein 2013) and how bacteria and viruses may be shaping the genomes and phenotypes of harbouring organisms (Gorski et al. 2003, Crochu et al. 2004, Degnan & Moran 2008, Keeling & Palmer 2008, Nikoh et al. 2008, Klasson et al. 2009, Rohwer et al. 2009, Holmes 2011, Rosario & Breitbart 2011, Ni et al. 2012, Reyes et al. 2012, Stern et al. 2012 , Horie et al. 2013, Husnik et al. 2013, Ioannidis et al. 2013, Seed et al. 2013 ). When we take into consideration the fact that there are one million bacteria and 10 million viral particles per millilitre of surface seawater (Suttle 2005, Ng et al. 2011b, Rosario & Breitbart 2011), maximising the NGS sequencing data generated by the *An. aquasalis* genome project becomes an opportunity to explore many of these new avenues. These vast surroundings and potentially associated microcosms may have left their mark upon the coevolving larval stages of this species while developing in brackish waters.
